# Supplementary material for: Compositional and predicted functional analysis of the gut microbiota of Radix auricularia (Linnaeus) via high-throughput Illumina sequencing
Source: PeerJ. 2018 Aug 28;6:e5537. doi: 10.7717/peerj.5537 (PMC6118204; doi:10.7717/peerj.5537)
Supplement: Supplemental Information 1 — A_1, A_2, A_3, A_4 represented adult snail samples, J_1, J_2, J_3, J_4 represented juvenile snails samples. [file peerj-06-5537-s001.docx]

| Samples | A_2 | A_1 | A_3 | A_4 | J_2 | J_3 | J_1 | J_4 |
| --- | --- | --- | --- | --- | --- | --- | --- | --- |
| Sequence number | 27905 | 37290 | 26823 | 30802 | 35172 | 27650 | 36526 | 28904 |
| Mean length | 433.21 | 435.60 | 434.36 | 435.90 | 432.40 | 433.98 | 436.43 | 433.24 |
| OTUs number | 861 | 908 | 807 | 943 | 911 | 885 | 934 | 877 |
