# Supplementary material for: Compositional and predicted functional analysis of the gut microbiota of Radix auricularia (Linnaeus) via high-throughput Illumina sequencing
Source: PeerJ. 2018 Aug 28;6:e5537. doi: 10.7717/peerj.5537 (PMC6118204; doi:10.7717/peerj.5537)
Supplement: Supplemental Information 2 — The core bacteria with abundance >0.5%. [file peerj-06-5537-s002.docx]

| Phylum | Class | Order | Family | Genus | OTU | Abundance (%) |
| --- | --- | --- | --- | --- | --- | --- |
| Tenericutes | Mollicutes | Mycoplasmatales | Mycoplasmataceae | norank | OTU585 | 6.157596 |
| Chloroflexi | Chloroflexia | Chloroflexales | Chloroflexaceae | unclassified | OTU107 | 4.385196 |
| Proteobacteria | Alphaproteobacteria | Rhodobacterales | Rhodobacteraceae | Paracoccus | OTU670 | 3.326138 |
| Proteobacteria | Gammaproteobacteria | Chromatiales | Chromatiaceae | Thiodictyon | OTU487 | 3.181956 |
| unclassified | unclassified | unclassified | unclassified | unclassified | OTU592 | 3.107475 |
| Cyanobacteria | Cyanobacteria | SubsectionIII | FamilyI | Microcoleus | OTU724 | 2.637889 |
| Firmicutes | Clostridia | Clostridiales | Lachnospiraceae | [Eubacterium]_rectale_group | OTU988 | 2.399312 |
| Cyanobacteria | Cyanobacteria | SubsectionII | FamilyII | Pleurocapsa | OTU358 | 2.350322 |
| Firmicutes | Clostridia | Clostridiales | Ruminococcaceae | Subdoligranulum | OTU669 | 1.530238 |
| Proteobacteria | Alphaproteobacteria | Rhizobiales | MNG7 | norank | OTU84 | 1.308788 |
| Fusobacteria | Fusobacteriia | o__Fusobacteriales | Leptotrichiaceae | unclassified | OTU278 | 1.235104 |
| Cyanobacteria | Cyanobacteria | SubsectionII | FamilyII | Pleurocapsa | OTU363 | 1.12876 |
| Firmicutes | Clostridia | Clostridiales | Ruminococcaceae | Faecalibacterium | OTU999 | 1.125175 |
| Chloroflexi | Caldilineae | Caldilineales | Caldilineaceae | norank_f__Caldilineaceae | OTU351 | 0.985773 |
| Cyanobacteria | Cyanobacteria | norank | norank | norank | OTU854 | 0.972629 |
| Chloroflexi | Chloroflexia | Chloroflexales | Chloroflexaceae | unclassified | OTU85 | 0.861904 |
| Cyanobacteria | Cyanobacteria | SubsectionIII | FamilyI | Microcoleus | OTU1170 | 0.844778 |
| Proteobacteria | Alphaproteobacteria | Rhodobacterales | Rhodobacteraceae | Rhodobacter | OTU828 | 0.762729 |
| Cyanobacteria | Cyanobacteria | norank | norank | norank | OTU1137 | 0.759941 |
| Proteobacteria | Gammaproteobacteria | Chromatiales | Chromatiaceae | Lamprocystis | OTU515 | 0.738434 |
| Proteobacteria | Alphaproteobacteria | Rhodobacterales | Rhodobacteraceae | Gemmobacter | OTU352 | 0.71135 |
| Actinobacteria | Actinobacteria | PeM15 | norank | norank | OTU127 | 0.697808 |
| Proteobacteria | Gammaproteobacteria | Enterobacteriales | Enterobacteriaceae | Kluyvera | OTU603 | 0.635276 |
| Actinobacteria | Actinobacteria | Micrococcales | Microbacteriaceae | Cryobacterium | OTU853 | 0.628903 |
| Proteobacteria | Gammaproteobacteria | Chromatiales | Chromatiaceae | unclassified | OTU503 | 0.609785 |
| Chloroflexi | Caldilineae | Caldilineales | Caldilineaceae | norank | OTU553 | 0.607395 |
| Cyanobacteria | Cyanobacteria | norank | norank | norank | OTU859 | 0.589871 |
| Cyanobacteria | Cyanobacteria | SubsectionIII | FamilyI | Leptolyngbya | OTU781 | 0.578718 |
| Cyanobacteria | Cyanobacteria | SubsectionIII | FamilyI | unclassified | OTU806 | 0.56199 |
| Cyanobacteria | Cyanobacteria | o__norank | norank | norank | OTU486 | 0.542076 |
| Actinobacteria | Actinobacteria | Pseudonocardiales | Pseudonocardiaceae | Actinomycetospora | OTU878 | 0.535305 |
| Proteobacteria | Alphaproteobacteria | Rhodospirillales | Rhodospirillaceae | Skermanella | OTU76 | 0.507424 |
| Proteobacteria | Gammaproteobacteria | Aeromonadales | Aeromonadaceae | Aeromonas | OTU578 | 0.506628 |
| Proteobacteria | Alphaproteobacteria | Rhodospirillales | Acetobacteraceae | Rubritepida | OTU87 | 0.500653 |
