# Supplementary figures and images for: Compositional and predicted functional analysis of the gut microbiota of Radix auricularia (Linnaeus) via high-throughput Illumina sequencing
Source: PeerJ. 2018 Aug 28;6:e5537. doi: 10.7717/peerj.5537 (PMC6118204; doi:10.7717/peerj.5537)

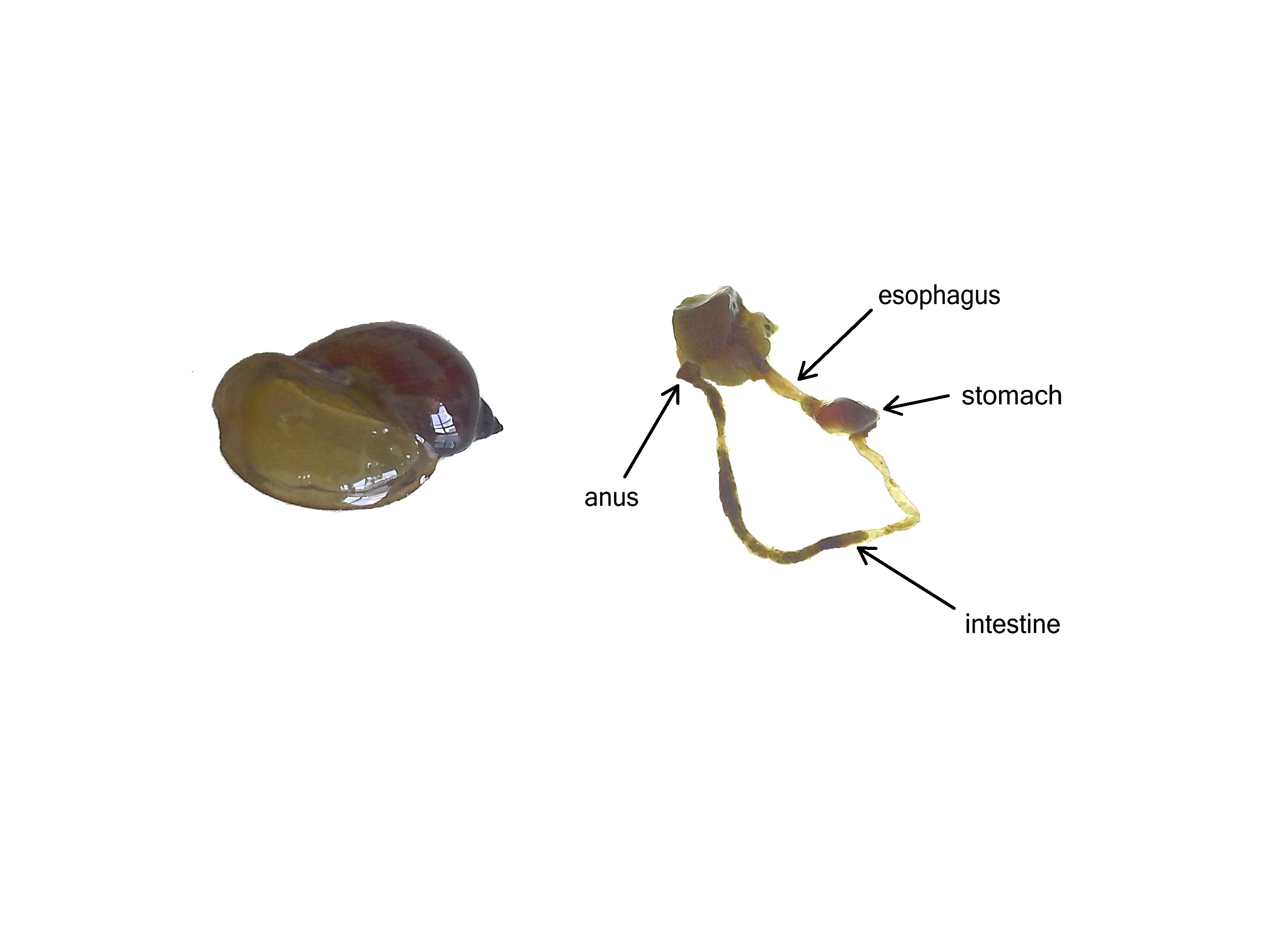

Supplement: Supplemental Information 4 — Photo credit: Zongfu Hu. [file peerj-06-5537-s004.png]

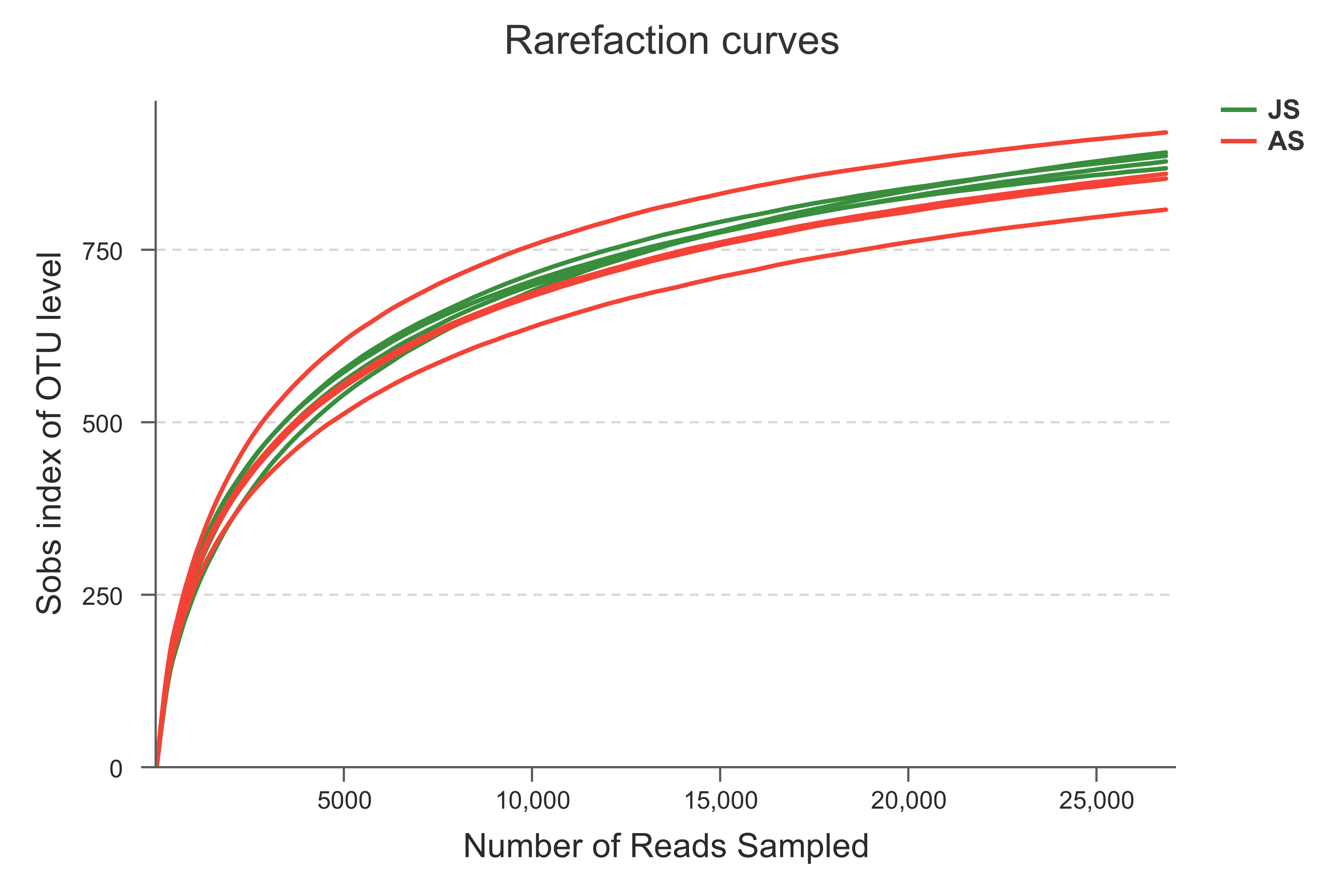

Supplement: Supplemental Information 5 — There were eight samples collected and successfully sequenced. Four samples were collected from juvenile snails and four samples from adult snails. Operational taxonomic units (defined at 97% sequence similarity) identified by Illumina Miseq sequencing of the V3-4 region of the 16S rRNA genes. [file peerj-06-5537-s005.png]

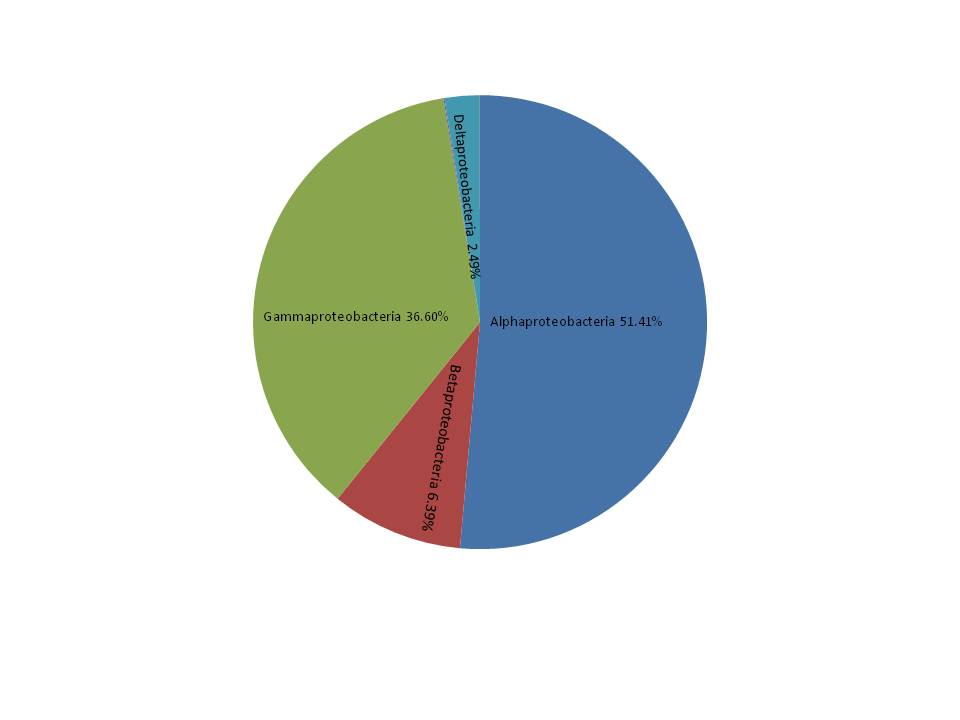

Supplement: Supplemental Information 6 [file peerj-06-5537-s006.jpg]

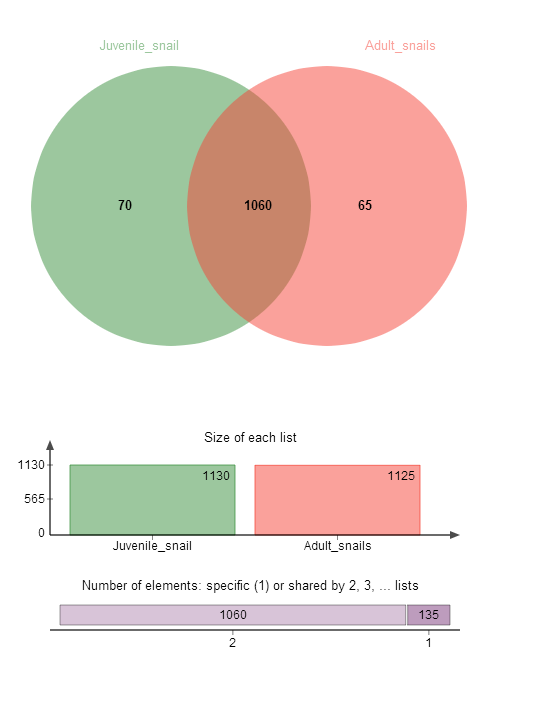

Supplement: Supplemental Information 7 [file peerj-06-5537-s007.png]

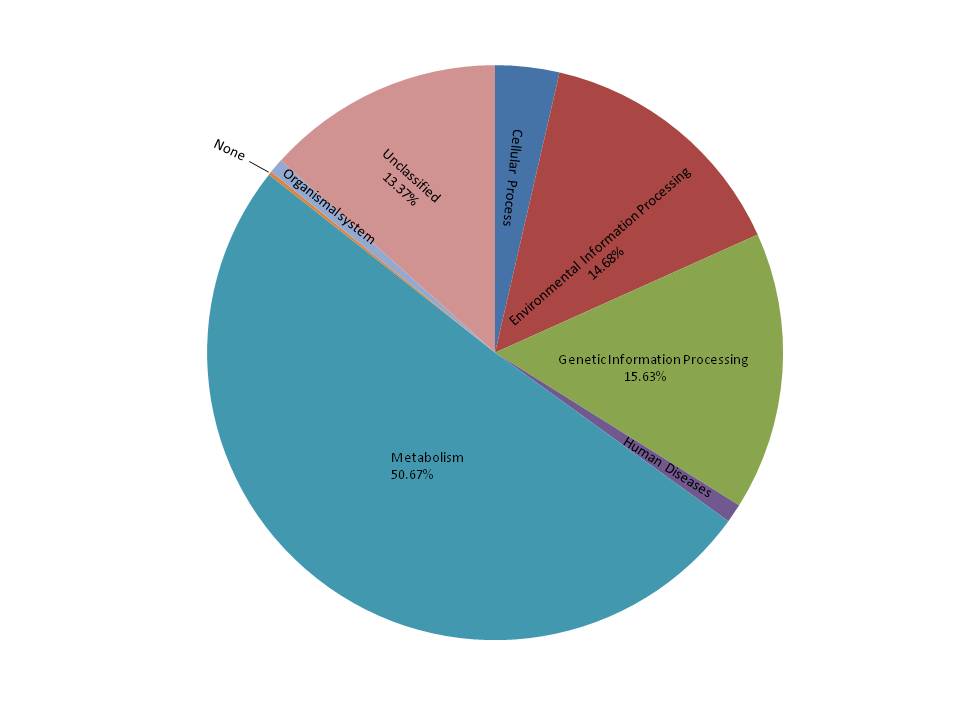

Supplement: Supplemental Information 8 [file peerj-06-5537-s008.jpg]

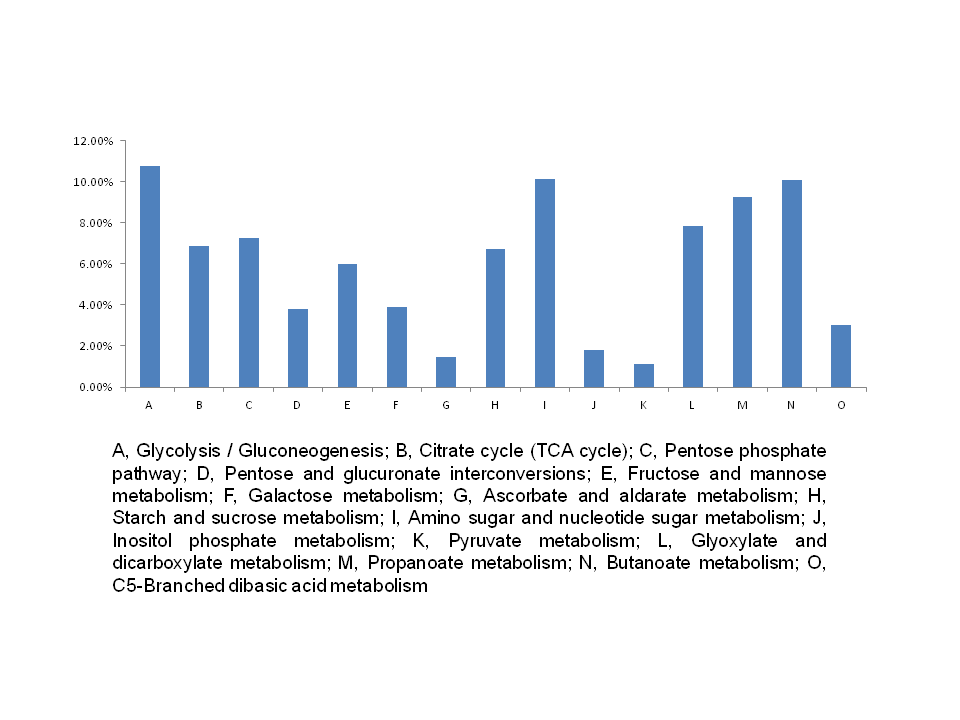

Supplement: Supplemental Information 9 [file peerj-06-5537-s009.png]

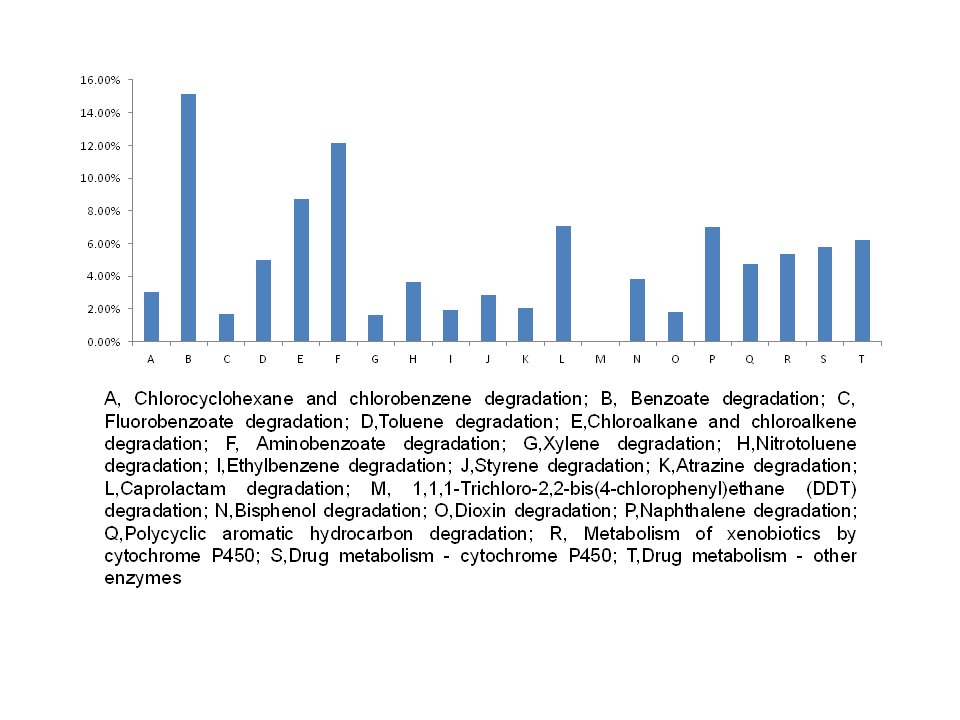

Supplement: Supplemental Information 10 [file peerj-06-5537-s010.png]
